# Quantifying variation in DLP‐ to‐ effective‐dose conversion factors in CT using a Monte Carlo‐based dose‐estimation software

**DOI:** 10.1002/acm2.70724

**Published:** 2026-08-30

**Authors:** Jelena M. Mihailovic, Renxin Chu, Da Zhang, Andrew M. Hernandez, Kai Yang, Lifeng Yu

**Affiliations:** ^1^ Department of Radiology Mayo Clinic Rochester Minnesota USA; ^2^ Department of Radiology University of Missouri Health Care Columbia Missouri USA; ^3^ Department of Radiology UVA Health University Hospital Charlottesville Virginia USA; ^4^ Department of Radiology Boston Children's Hospital Boston Massachusetts USA; ^5^ Department of Radiology UC Davis Health Sacramento California USA; ^6^ Department of Radiology Massachusetts General Hospital Boston Massachusetts USA

**Keywords:** computed tomography, effective dose, effective dose conversion factors, uncertainty analysis

## Abstract

**Background:**

A common method to estimate the effective dose in CT is to multiply the dose length product in a CT scan by a conversion factor (k). The k‐factors are determined by dividing the effective dose (calculated using a weighted summation of organ doses) by the dose length product (DLP) for specific digital phantoms. The k‐factors determined in this way may vary substantially depending on the effective dose calculation methods and phantom models used.

**Purpose:**

The purpose of this study was to estimate variation associated with different acquisition and patient characteristics in the quantification of k‐factors.

**Methods:**

K‐factors were calculated using VirtualDoseCT for a diverse set of computational phantoms representing different patient models, scanner models, and seven CT exams. The tube potential varied from 80 to 120 kV. The scan length was varied by ± 5 cm relative to the default setting. The CTDIvol was set to 10 mGy when tube current modulation was not used; otherwise, the software default was used. A multivariable regression method was employed to derive an empirical formula for k‐factors, along with model‐uncertainty analysis and Sobol's sensitivity analysis to identify the influence of each variable.

**Results:**

K‐factors for all exams increased with kV. Negative dependence of k‐factors on BMI was observed, with lower values in males than in females. Moderate to high variation was observed in the quantification of k‐factors without TCM, which remained unchanged with TCM. Based on Sobol's indices (0.8–1), k‐factors were influenced most by scan length and patient size for adults, and scan length and age for pediatric phantoms.

**Discussion:**

The study showed that a large spread in k‐factors was predominantly influenced by scan length, patient size and age. The heterogeneity in k‐factors may lead to large heterogeneity in the effective dose estimate if the DLP×k method is used. These results indicate that age and size specific k‐factors to estimate effective dose from DLP may be warranted.

**Conclusions:**

A set of k‐factors should be established to account for these factors and improve effective dose estimations.

## 1 INTRODUCTION

Radiation dose and the potentially associated risk from computed tomography (CT) remain an critical topic in the field of medical physics.^1^ Considering the noteworthy increase in the number of CT exams, the accuracy of individual CT exam doses continues to impact the total dose estimation to the public from medical procedures involving ionizing radiation.^2^

The volume CT dose index (CTDI_vol_) and dose‐length product (DLP) are standardized indices of radiation dose output from the scanner, based on two standardized phantoms (acrylic cylinders with a diameter of 16 and 32 cm), and are reported for each CT exam. CTDI_vol_ is a measure of scanner radiation output in terms of dose to the phantom but does not account for different patient sizes. DLP (CTDI_vol_ x scan length) provides a surrogate that is approximately proportional to the total energy absorbed in the standardized phantom, attributable to the complete scan acquisition.^3^ Although CTDI_vol_ and DLP provide standardized metrics quantifying the radiation dose output from the scanner, they do not directly quantify the dose to the patient or each specific organ, nor the risk of health detriment.^3^ A widely used metric in CT practice to quantify risk is effective dose (*E*), which is calculated as a weighted sum of equivalent doses in tissues and organs, where the weighting factor is determined by the relative radiation‐induced biological risk of each organ.^4^ Effective dose provides an estimate of the whole‐body equivalent dose that would have a similar risk as that in a partial body exposure. It allows comparison of radiation risk among different types of radiation and examinations. To estimate the effective dose, a direct approach is to follow the definition of effective dose, that is to determine all the organ doses and then perform a weighted summation. Measurement of organ doses is not trivial; therefore, many rely on Monte Carlo simulation based on stylized anthropomorphic or patient‐specific digital phantoms.

Many software packages have been developed for organ dose and effective dose estimates over the past two decades, including Monte Carlo simulation of the radiation transport in the body by Zankl et al,^5^ revised E/DLP coefficients and voxel phantoms,^6^ VirtualDose,^7^ CT‐Expo,^8^ NCICT,^9^ among others. Different software packages use different computational phantoms (stylized, anthropomorphic, or patient‐specific) and boundary representation phantoms (BREP). It has been reported that the lack of anatomical realism in the stylized phantoms results in differences in radiation dose when compared to a real patient.^10^ Different packages, including VirtualDose^7^, CT‐Expo^8^ and National Cancer Institute dosimetry system for Computed Tomography (NCICT)^9^ also vary in dose desriptors and the availability of CT scanner parameters. Studies have shown discrepancies of about > 200% when estimating the effective dose.^11^, ^12^, ^13^

For convenience, especially when organ doses, based on Monte Carlo simulations, were not available, a simplified method was proposed and has been widely used to estimate the effective dose for patient exams in CT. This method estimates the effective dose by multiplying the DLP by a conversion factor (k), which is dependent on the scanned anatomical region.^14^ Originally, k factors were developed using stylized computational phantoms.^6^ Under European Guidelines^15^ they were modified to include pediatric patients and extended scan regions. The ICRP publication 102^16^ and AAPM report 96^17^ provided k‐factors for adults of standard sizes and pediatric patients of 0, 1, 5, and 10 years of age for the most common CT exam types (head, neck, head and neck, chest, abdomen and pelvis, trunk). The published k factors did not account for sex, age, patient size, and were derived using hermaphrodite stylized human phantoms based on previous ICRP recommendations. Later developments are based on the tissue weighting factors published by the International Commission on Radiological Protection (ICRP) Publication 60^18^ and updated ICRP Publication 103^19^ voxel‐based, age‐specific pediatric and adult phantoms, improving accuracy for different body sizes and Monte Carlo transport calculations. The major limitation of using k‐factors is their dependency on many factors, such as phantom diameter, tube potential, scanning length, and so forth.^20^ These previous studies have shown that the effective dose estimated using fixed, region‐specific k‐factors can simplify assessments of radiation exposure in real clinical settings. The dependence of the effective dose on patient, protocol, and scanner‐related parameters drives the need for empirical models. Recently, more size‐specific k‐factors in CT for different anthropomorphic computational phantoms have been studied, but still, there are no definitive results on their accuracy.^21^ Also, recent research involves calculating distinct k‐factors for dual‐energy CT, acknowledging that standard conversion factors are inadequate for advanced scanner geometries and dual‐energy CT.^22^

The goal of this study was to investigate how using size and protocol‐specific k‐factor affects the effective dose estimate across a diverse patient population. Different contributing factors are explored, and differences in obtained k‐factors for different CT acquisition parameters and patient characteristics were quantified. Also, we provided empirical formulas for k‐factor assessments.

## 2 METHODS

A radiation dose tracking and reporting software tool, VirtualDoseCT (Virtual Phantoms Inc.)^7^ was employed in this study with computational phantoms developed at the Rensselaer Polytechnic Institute and the University of Florida.^7^ Estimation of effective dose was based on organ dose estimates from Monte Carlo simulations with tissue weighting coefficients as specified in the ICRP Publication 103.^19^ A total of twenty‐five computational phantoms were used to simulate patients of different genders and sizes, represented by body mass index (BMI (kg/m^2^)) for adults and by age (0, 1, 5, 10, and 15 years) for pediatric phantoms. There are ten adult phantoms with different weights to describe the population, grouped into five BMI categories according to the World Health Organization (WHO) classification: normal weight, overweight, obese 1, obese 2, and obese 3. Details about the BMI group range, weight, and height for different adult and pediatric patient phantoms are explained in detail elsewhere.^23^ Briefly, the height for male phantoms is always the same, 176 cm, and for females, 156 cm. Only the weight was changed to meet the average of the WHO BMI categories (Table 1).

**TABLE 1:** Patient size groups based on BMI (kg/m^2^) for adult phantoms (adapted from Huo et al.^23^).

Gender	Normal weight	Overweight	Obese level 1	Obese level 2	Obese level 3
Male	23.5	27.7	33.3	37.8	45
Female	23.9	28.3	34.1	38.5	46.4

Our study included the most commonly performed scan regions: head, neck, chest, abdomen, pelvis, and the combined abdomen/pelvis (AP) and chest/abdomen/pelvis (CAP) regions. The default scan length in VirtualDose, hereafter defined as “reference scan length,” differed between males and females. More details can be found in the study by Liang et al.^24^ The scan length varied by ± 5 cm in the z‐direction relative to the reference scan length. Besides the mentioned parameters, tube voltage was varied from 80 to 120 kV (in 20 kV intervals). First, we did dose estimation without TCM. The CTDI_vol_ was fixed at 10 mGy (16 cm CTDI phantom for head and neck, 32 cm CTDI phantom for the other exam types), the pitch was set to 1, and other parameters are provided in Table 2. Given that most exams in clinical practice are performed with TCM, we also performed dose estimation with TCM (Table 3). VirtualDose includes a range of CT scanner models from different manufacturers. Three CT scanner models from three different manufacturers were used in this study, including Siemens (Somatom Force), GE (Discovery 750HD), and Philips (Ingenuity series). In VirtualDose, the user can select Yes for tube current modulation, which indicates that TCM is in use. That requires inputting the average mAs per rotation for the entire scan rather than a constant value. The software will calculate doses using normalized CTDIw values combined with the average mAs and pitch. The software adjusts calculations based on the assumption that TCM modulation tube current to maintain uniform noise levels.^7^ In Table 3, the CTDIvol range shows a difference across the kV values used in our study and is normalized for k‐factor calculation. It is noteworthy to say that in clinical scanners, modulation strength is vendor‐specific. For the Siemens system (CAREDose4D), the operator selects weak, average, strong, or very strong adaptation, which correspond quantitatively to increasing values of the control parameter α. For the GE system (AutomA/Smart mA), tube current modulation was determined using a paradigm with constant noise and no strength setting. K factors were then calculated as the ratio of E and DLP (E/DLP) in units of mSv/mGy·cm. As mentioned above, the CTDI_vol_ and DLP values were based on a 16 cm reference phantom for head and neck exam types and a 32 cm reference phantom for chest, abdomen, pelvis, AP, and CAP exam types.

**TABLE 2:** Parameters used for each CT exam type without TCM for adult and pediatric phantoms.

Exam type	kV	Pitch	Bowtie filter	CTDI_vol_ (mGy)	Collimation (mm) (Siemens)	Collimation (mm) (GE)	Collimation (mm) (Philips)
Head	80,100,120	1	Head	10	38.4	40	40
Neck	80,100,120	1	Head	10	38.4	40	40
Chest	80,100,120	1	Body	10	57.6	40	40
Abdomen	80,100,120	1	Body	10	57.6	40	40
Pelvis	80,100,120	1	Body	10	57.6	40	40
AP	80,100,120	1	Body	10	57.6	40	40
CAP	80,100,120	1	Body	10	57.6	40	40

**TABLE 3:** The range of parameters used for different CT exam types with TCM for adult and pediatric phantoms.

Exam type	kV	Pitch	Bowtie filter	Collimation (mm) (Siemens)	Collimation (mm) (GE)	Collimation (mm) (Philips)	CTDI_vol_ (mGy) (Siemens)	CTDI_vol_ (mGy) (GE)	CTDI_vol_ (mGy) (Philips)
Head	80,100,120	1	Head	38.4	40	40	4–14	6–16	3–12
Neck	80,100,120	1	Head	38.4	40	40	4–14	6–16	3–12
Chest	80,100,120	1	Body	57.6	40	40	1–6	2–8	1–7
Abdomen	80,100,120	1	Body	57.6	40	40	1–6	2–8	1–7
Pelvis	80,100,120	1	Body	57.6	40	40	1–6	2–8	1–7
AP	80,100,120	1	Body	57.6	40	40	1–6	2–8	1–7
CAP	80,100,120	1	Body	57.6	40	40	1–6	2–8	1–7

### 2.1 Multivariable linear regression analysis

Multivariable linear regression was performed to quantify the relationship between all variables included in this study, and to provide empirical formulas for the calculation of k‐factor based on linear regression coefficients, following Equation 1 for adults and Equation 2 for pediatric phantoms.

(1) $$\begin{array}{cc} k-\mathrm{fact}\mathrm{ors} & =\mathrm{kc}+a\times \mathrm{kV}+b\times \mathrm{BMI}+c\times \mathrm{Gend}\mathrm{er}\\ & \;+d\times \mathrm{Scan}\;\mathrm{Leng}\mathrm{th}+e\times \mathrm{Vend}\mathrm{or} \end{array}$$

(2) $$\begin{array}{cc} k-\mathrm{fact}\mathrm{ors} & =\mathrm{kc}+a\times \mathrm{kV}+b\times \text{Age}+c\times \text{Gender}\\ & \;+d\times \text{Scan Length}+e\times \text{Vendor} \end{array}$$

### 2.2 Uncertainty and sensitivity analysis

While multivariate linear regression analysis provides empirical formulas for the k‐factor, it does not quantify the potential error of variation in a model's output due to uncertainties in input data.^25^ As an additional step, uncertainty and sensitivity analyses were performed to approximate overall variations in k‐factor results that depend on the combined effect of multiple variables of interest. All statistical analyses were performed using the R software package v.4.2.2 (R Core Team, 2023, Vienna, Austria).

The task of the multivariate uncertainty method was to propagate the uncertainty estimates through a data reduction equation by simultaneously computing the uncertainty estimates for each variate, along with the correlation between them. Ultimately, this approach provides an output vector containing all input variates.^26^, ^27^ The more the fit deviates from linearity, the higher the uncertainty is.

Finally, using Sobol's method for sensitivity analysis, the amount of variation in the output was determined and then attributed to variations in each input variable, considering their potential correlation.^28^ Sobol's method^29^, ^30^, ^31^, ^32^ is based on the decomposition of the model output variance into a sum of variances of the input parameters in increasing dimensionality. With this method, the contribution of each input parameter and its interactions to the overall model output variance can be determined. Briefly, each input parameter *m* (*m* = 1, 2,…, *n*) is considered to be in a range over some finite interval. The sensitivity of the model output to the input parameters is then assessed as a function of m “*f(m)*”. Under the probabilistic interpretation of the parameters, *f(m)* is a random variable with mean *f(0)* and variance V^32^. Decomposition of the V measures the fraction of the total output variance that can be attributed to the uncertainty in a single input variable alone (first‐order Sobol indices) or interaction between two input variables (second‐order Sobol indices). This is done first by decomposing *f(m)* into:

(3) $$\begin{array}{cc} f\left(m\right)=f\left(0\right)+\sum_{r=1}^{x}f_{r}\left(m_{r}\right) & +\sum_{r=1}^{x}\sum_{r\neq j}^{x}f_{rj}\left(m_{r},m_{j}\right)\\ & \;+..f_{1\text{…}x}\left(m_{1},m_{2},\text{…}m_{x}\right) \end{array}$$

Integration of both sides of Equation 3 leads to:

(4) $$V=\sum_{r=1}^{k}V_{r}+\sum_{r<j}V_{rj}+\sum_{r<j<l}V_{rjl}+\cdots +V_{1,2\text{…}k}$$

Finally, Sobol's sensitivity for the subset of the parameters is:

(5) $$S_{r1\text{…}rx}=\frac{V_{r1\text{…}rx}}{V}$$

In this study, first‐order sensitivity as the main effect is used to measure the fractional contribution of a single parameter to the output variance and is defined using Sobol's indices (S) with a range of 0 to 1. The higher the Sobol's indices (*S*) are the more influential the variable is. For a multivariable system, it is customary to use a threshold of *S* = 0.1 to determine how significant the influence of individual input variables is on the model output.

## 3 RESULTS

**FIGURE 1:**
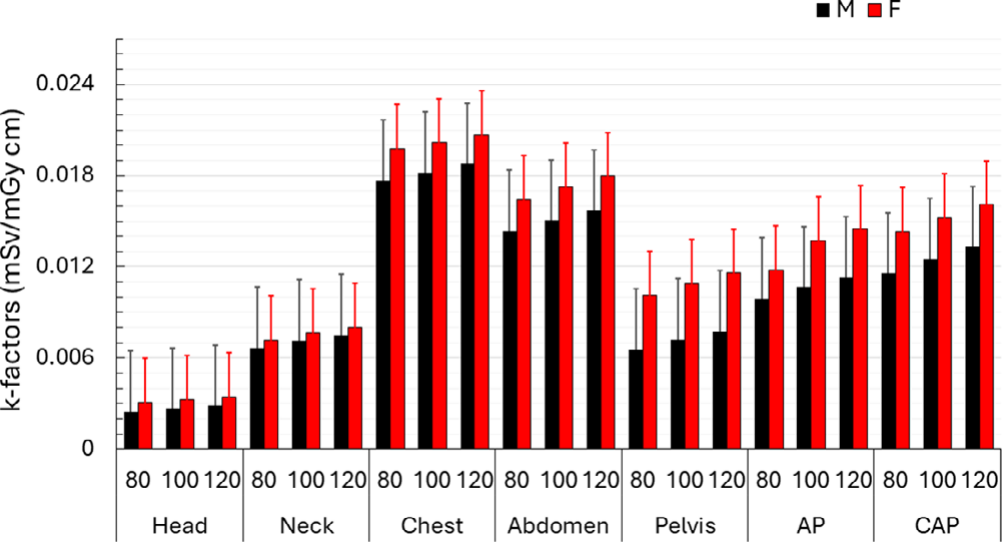
Dependence of k‐factors on tube voltage for adult CT exams without TCM, with the standard deviations.

For all adult CT exams, k‐factors increased with tube voltage. The highest increase was seen for the adult chest and adult abdomen exams, followed by the pelvis, AP, and CAP (Figure 1).

**FIGURE 2:**
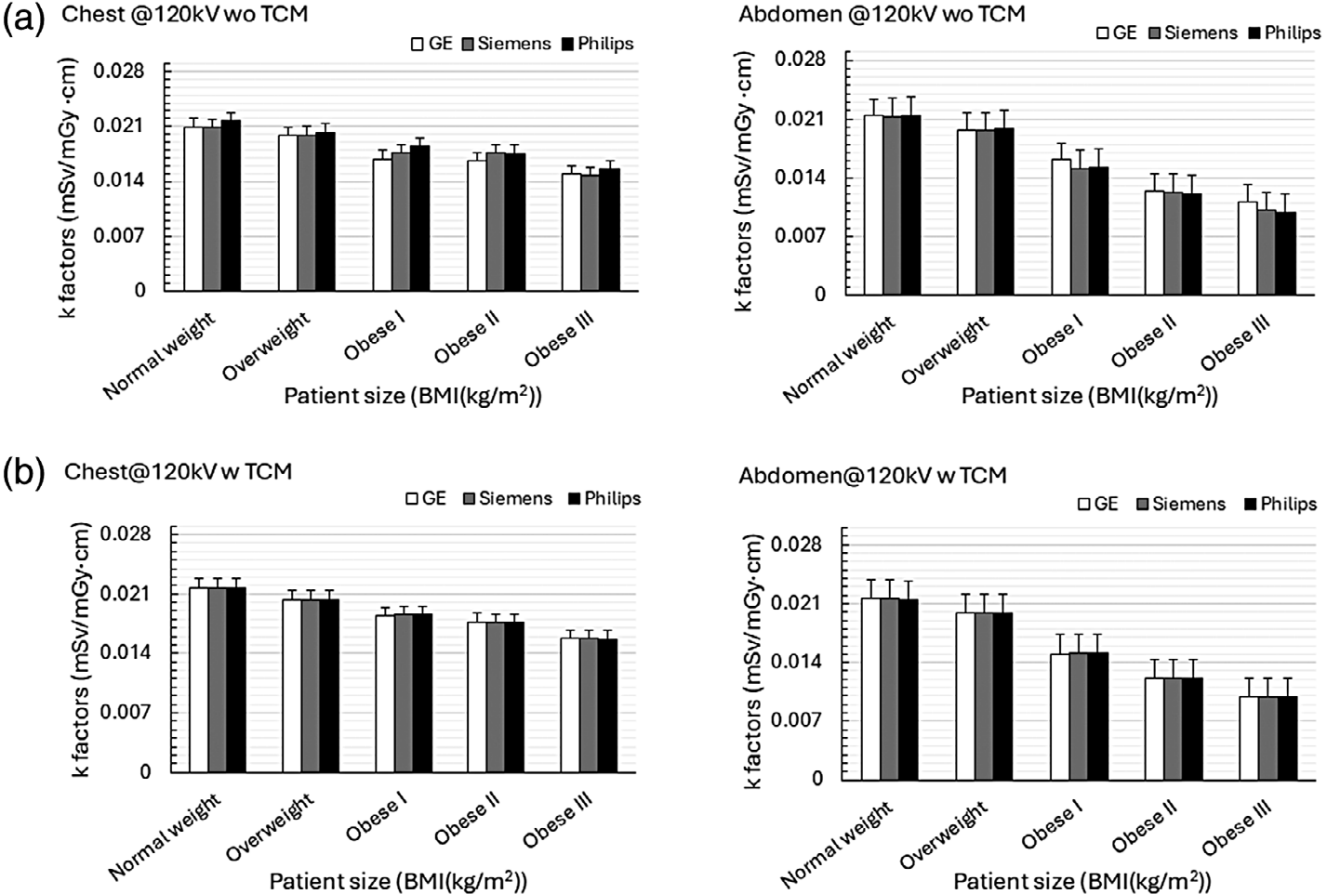
Dependence of k‐factors for chest and abdomen exam types at 120 kV on patient size for males (a) without TCM and (b) with TCM for three CT vendors.

**FIGURE 3:**
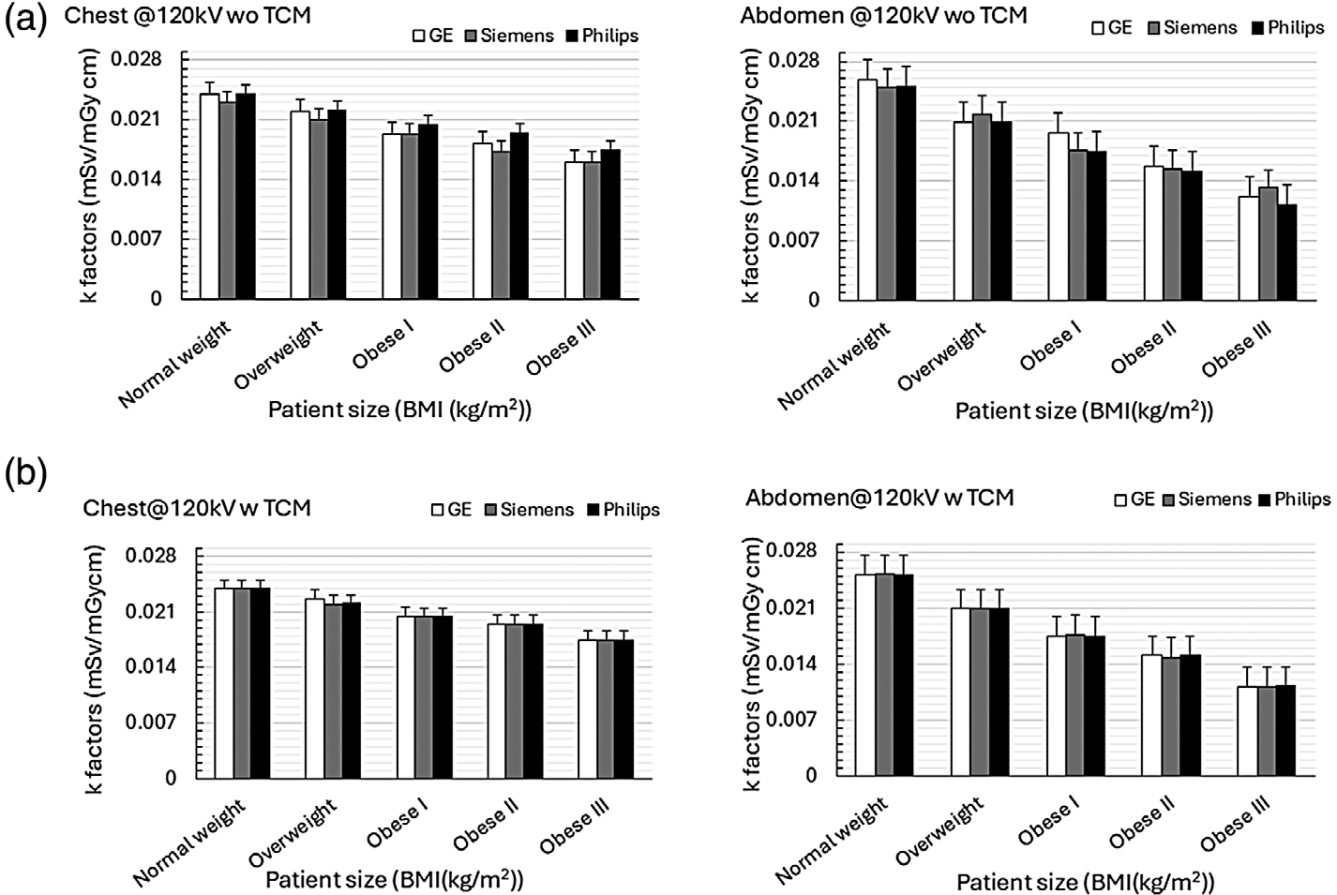
Dependence of k‐factors for chest and abdomen at 120 kV on patient size for females without (a) and with TCM (b) for three CT vendors.

There was a noted difference of about 13–18 % in k‐factors between male (black bars) and female (red bars). For all adult exams, an increase in k factors with increased kV was statistically significant (*p* < 0.05, see Appendix A). With TCM, the same results were seen. K‐factors increased with an increase in kV (*p* < 0.05), with a difference in k‐factor values between male and female phantoms. Examples of k‐factors for chest and abdomen exams at 120 kV without and with TCM and for males and females are shown in Figures 2 and 3, respectively.

It appears that for both genders in the settings without TCM, going from normal to obese level 3 patient size group, k‐factors decrease, regardless of the CT scanner models, with a negative regression slope (Table 4), indicating an inverse relationship between k‐factors and patient size (Figures 2a and 3a). This decrease in k‐factors with patient size showed a statistically significant difference (*p* < 0.05) for all exams except head. Similar results, with similar negative regression slopes (Table 5), were seen in the setting with TCM for males (Figure 2b) and females (Figure 3b), with *p* < 0.05.

**TABLE 4:** Regression coefficients from the regression analysis of dependents of the k‐factors on patient size for chest and abdomen, without TCM.

	Chest			Abdomen		
	GE	Siemens	Philips	GE	Siemens	Philips
Male	−0.0015	−0.0014	−0.0015	−0.0028	−0.0030	−0.0031
Female	−0.0020	−0.0018	−0.0015	−0.0033	−0.0030	−0.0034

**TABLE 5:** Regression coefficients from the regression analysis of the dependence of the k‐factors on patient size for chest and abdomen, with TCM.

	Chest			Abdomen		
	GE	Siemens	Philips	GE	Siemens	Philips
Male	−0.0015	−0.0015	−0.0015	−0.0031	−0.0031	−0.0031
Female	−0.0016	−0.0015	−0.0015	−0.0034	−0.0034	−0.0034

**FIGURE 4:**
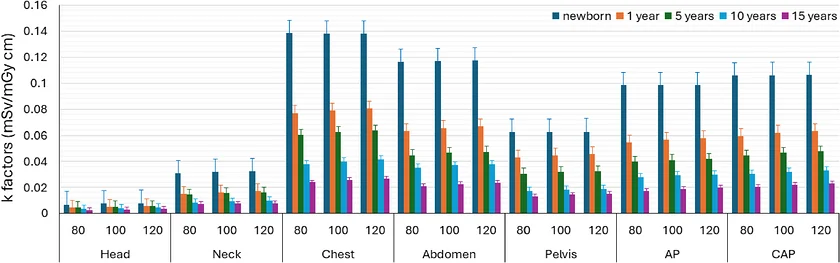
Dependence of k‐factors on tube voltage for pediatric CT exams without TCM.

In pediatric phantoms, the k‐factors increased across all tube potentials for the chest, abdomen, CAP, AP, pelvis, neck, and head (Figure 4).

There was no difference between genders, which was seen for adult phantoms. Going from the newborn age group (dark blue bars), k factors tend to decrease towards older age, being the lowest for 15 years (purple bars), with negative regression coefficients. In the setting without TCM, there was a statistically significant difference in k‐factors for pelvis, AP, and CAP (*p* < 0.05), while with TCM, there was no statistically significant difference (*p* > 0.05) for any of the explored exams.

Empirical formulas with regression coefficients from multivariable linear regression analysis and distribution of k‐factors from this study for adult and pediatric exams are provided in Appendix A. A violin plot of estimated k‐factors across the whole study showed a considerable range of values for adult and pediatric models for chest, abdomen, pelvis, AP, and CAP, suggesting a large dispersion of k‐factor values (Figures S1 and S2).

**FIGURE 5:**
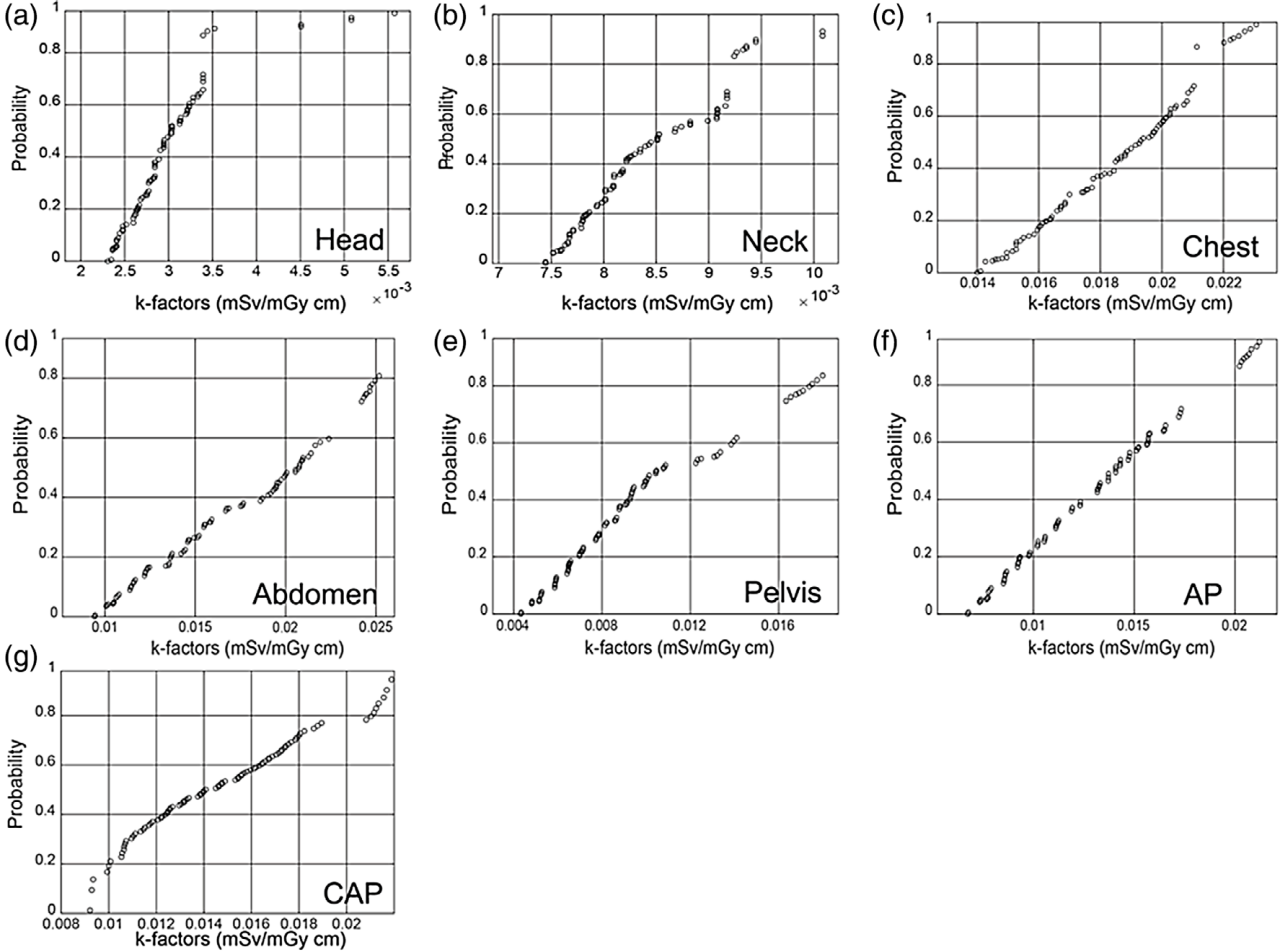
Propagation of the probability in uncertainty analysis for adult exams without TCM.

Uncertainty analysis showed moderate to high deviation for all adult exams without TCM (Figure 5a–g).

**FIGURE 6:**
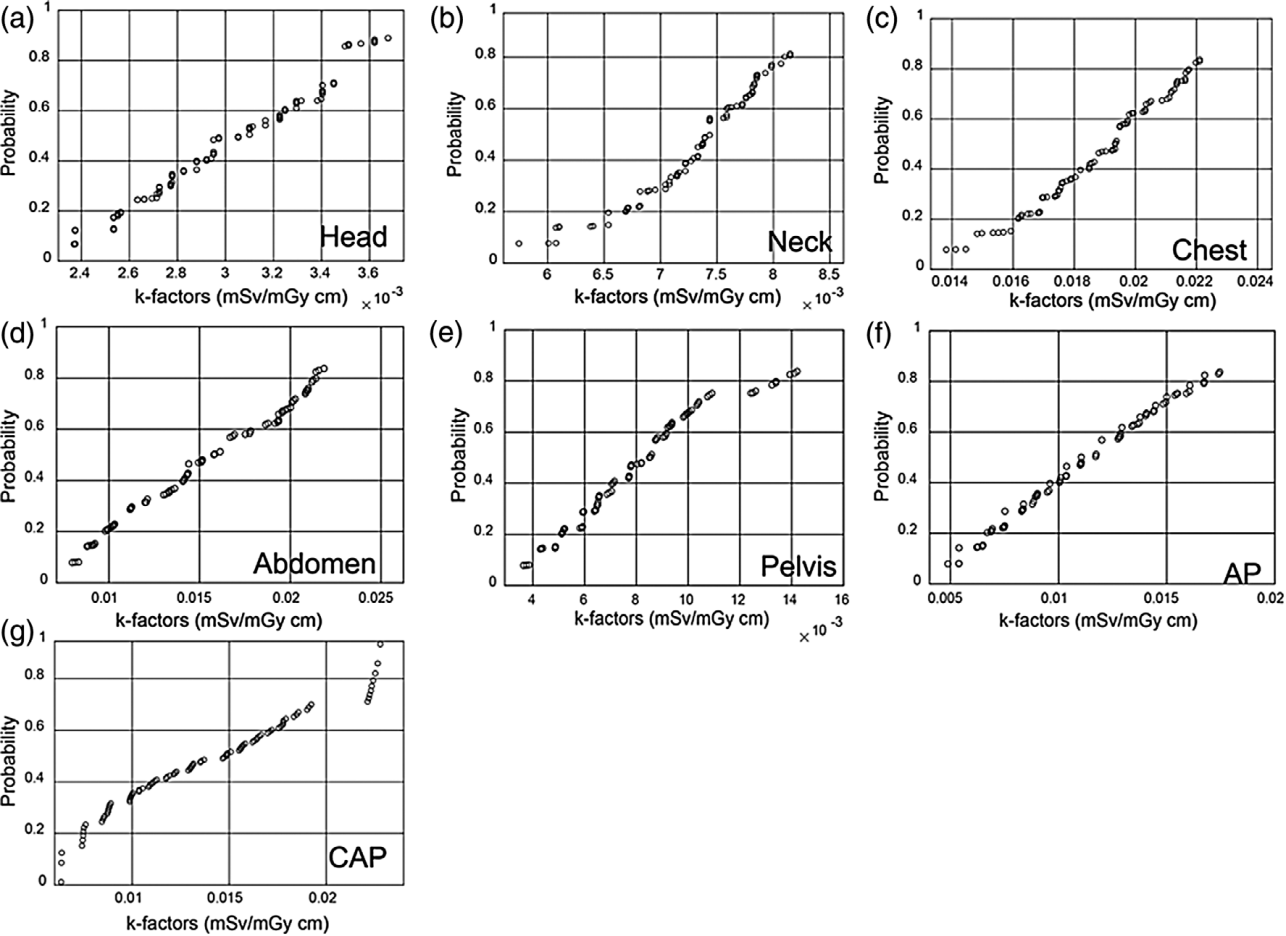
Propagation of the probability in uncertainty analysis for adult exams with TCM.

Compared to the analysis with TCM (Figure 6a–g), it appears there was an improvement only for the head and neck exam, reflected in a fit that more closely followed a linear trend. Other exam types did not show improvement when TCM was used.

**FIGURE 7:**
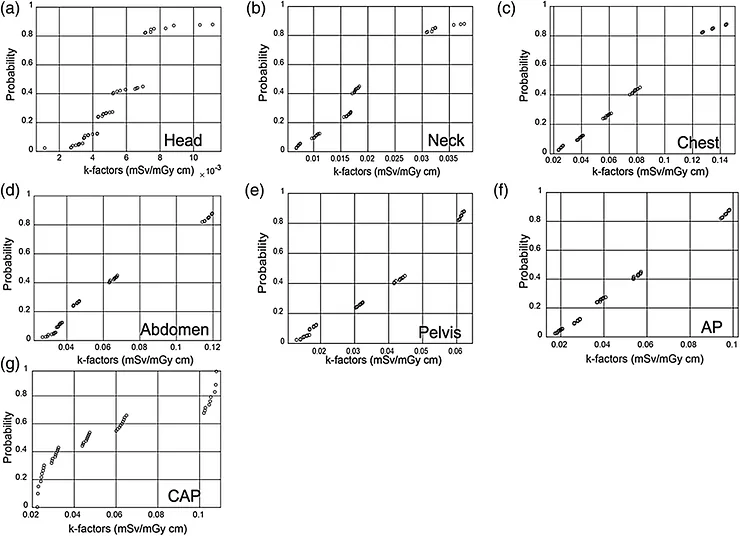
Propagation of the probability in uncertainty analysis for pediatric exams without TCM.

**FIGURE 8:**
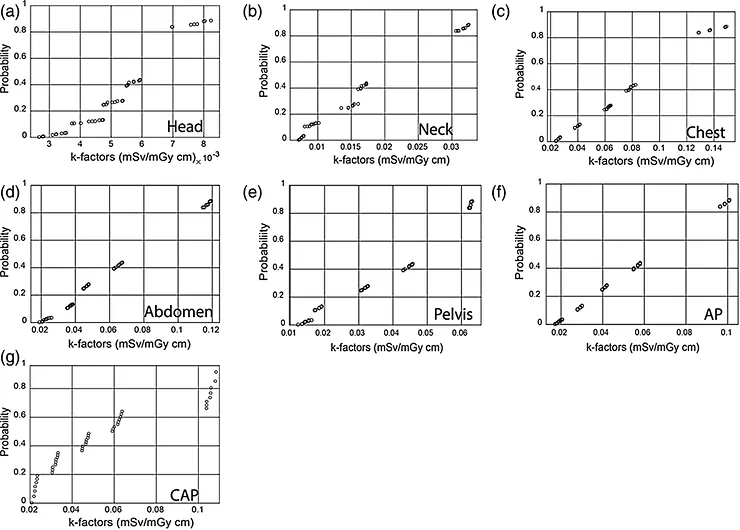
Propagation of the probability in uncertainty analysis for pediatric exams with TCM.

For pediatric phantoms, moderate dispersion was seen in all exams without TCM (Figure 7a–g), which was slightly improved for head exams with TCM (Figure 8a–g), but no significant changes were observed in other exams.

**FIGURE 9:**
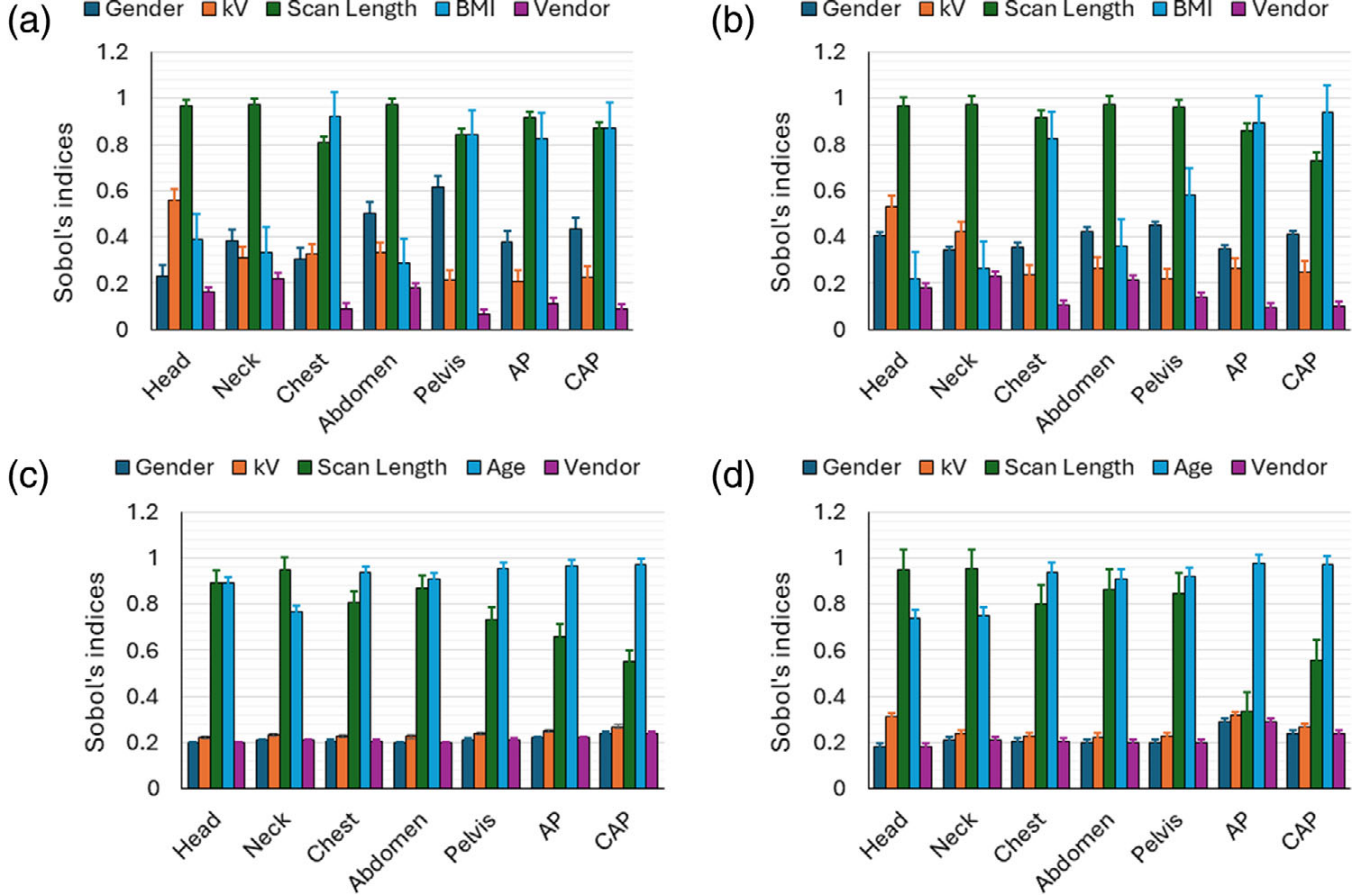
Sobol's indices for adult phantoms without (a) and with (b) TCM, respectively, and pediatric phantoms without (c) and with TCM (d).

Sensitivity analysis showed that, within a given range of variables, observed differences in k‐factors when TCM was not used were most influenced by scan length and patient size for adults (Figure 9a, green and light blue bars, respectively), with Sobol's indices: *S* = 0.8–1. The lesser influence was observed for gender (Figure 9a, dark blue bar), with *S* = 0.4–0.7, and vendor (Figure 9a, purple bar), with *S* = 0.2–0.5. For pediatric phantoms, scan length and age (Figure 9c, green and light blue bars, respectively) have the biggest impact on k‐factor inconsistencies, *S* = 0.8–1. No difference was found when TCM was used for both adult and pediatric phantoms (Figure 9b,d, respectively).

Compared with the results from ICRP102/AAPM Report 96^17^ differences for some exams are over 200% (Table 6), which leads to a significant under/overestimate of the patient's effective dose.

**TABLE 6:** Comparison of k‐factors between our study and ICRP 102/AAPM Report 96 for different CT exams at 120 kV.

Age (years)		Head	Neck	Chest	AP	CAP
0	our study	0.0081	0.0324	0.1380	0.0985	0.1062
	AAPM 96	0.0110	0.0170	0.0390	0.0490	0.0440
	**Difference (%)**	**−26.47**	**90.85**	**253.81**	**100.92**	**141.37**
1	our study	0.0057	0.0172	0.0806	0.0579	0.0634
	AAPM 96	0.0067	0.0120	0.0260	0.0300	0.0280
	**Difference (%)**	**−14.34**	**43.48**	**209.97**	**93.09**	**126.30**
5	our study	0.0059	0.0162	0.0636	0.0419	0.0477
	AAPM 96	0.0040	0.0110	0.0180	0.020	0.0190
	**Difference (%)**	**46.363**	**47.04**	**253.27**	**109.40**	**150.85**
10	our study	0.0047	0.0098	0.0413	0.0300	0.0331
	AAPM 96	0.0032	0.0079	0.0130	0.0150	0.0140
	**Difference (%)**	**47.90**	**23.63**	**217.98**	**100.18**	**136.57**
Adult^*^	our study	0.0032	0.0078	0.0197	0.0129	0.0147
	AAPM 96	0.0021	0.0059	0.0140	0.0150	0.0150
	**Difference (%)**	**50.14**	**31.60**	**40.91**	**−14.14**	**−2.06**

* Since in the AAPM Report 96, there are no k‐factors specified for different patient sizes, we used the average value for adult patients for comparison.

## 4 DISCUSSION

The accuracy of effective dose estimations using k‐factors can only be determined when the full range of variation is known. In this study, patient‐level data were created simulating different clinical conditions to map out the effective dose under these conditions. Study highlights a significant spread in the calculated k‐factor values, on factors such as the exam type, tube voltage, patient size, age, scan length, and gender. Commercially available software, VirtualDose, was used to better understand differences in derived k‐factors associated with different patient and scanner‐related parameters and their influence on effective dose estimates. The study showed the k‐factors were significantly affected by BMI for adult and age for pediatric phantoms, amongst other parameters. Also, empirical formulas are used to estimate k‐factors, serving as a reference to existing k‐factors (e.g., AAPM Report 96) in the literature, and could be used in scenarios when an estimate of the effective dose is required, but superior tools for organ dose calculations are not readily accessible. By counting variations and the main variable dependencies, it would enable an effective dose assessment under the conditions used in this study.

As tube voltage increased, the k‐factors increased for all adult and pediatric exams, which is consistent with results from similar studies.^10^, ^14^, ^33^ However, the sensitivity analysis in this study revealed that tube voltage had only moderate first‐order Sobol indices, suggesting that while it is not the dominant factor, its influence on k‐factors should not be overlooked.

For adult phantoms, there was a difference in k‐factors based on gender, making it a noteworthy factor in the discrepancies seen with their moderate S values. This difference could result from the default scan length in the z direction for male versus female phantoms in the VirtualDose tool, which involves the breast and gonads. For pediatric phantoms, the scan lengths for males and females were not different by default, so gender variation (low S) did not significantly affect the results as it did in adult phantoms. The same behavior was observed with and without the TCM. While the result showed large uncertainties based on Sobol's indices, it must be taken with caution. Although the predictors for this study were considered based on their clinical importance and their independence was considered, some degree of collinearity might still have influenced coefficient estimates.

For adult and pediatric phantoms and CT exams analyzed in this study, scan length was a significant variable and appeared to contribute to the spread of the data observed in the output model, both with and without TCM (Figure 9a–d). Another variable that greatly impacted the observed k‐factors spread was patient size, especially for chest, pelvis, AP, and CAP exams. Although TCM showed slightly lower Sobol indices for head, chest, and pelvis exams, this change was not statistically significant. The decrease in k‐factors with increasing patient size, particularly for chest, pelvis, AP, and CAP exams, was similar to findings by Liang et al.^24^ confirming that weight plays an important role, although the study by Li et al.^34^ also demonstrated differences in k‐factors when using obese patients of varying heights. Significant differences between exam types were not observed without and with TCM, although the literature^35^ suggests a decrease in k‐factors for chest exams with increasing patient size for both male and female adult models. This disparity could be due to the different methodology used by Sookpeng et al.^35^ Nonetheless, applying k‐factors estimated for average patients to larger BMI groups could introduce inaccuracies. For pediatric phantoms, age significantly affected the overall spread of the data across most exams with and without TCM, consistent with findings from Chu et al.^36^ Different CT manufacturers were included in this study to better assess their potential contribution to the spread in k‐factor values. Different CT manufacturers use different geometries and acquisition techniques. Beam quality, filtration, and collimation, as shown in this study, could be different for different CT scanners, affecting effective dose estimates. The main difficulty is to model different kinds of TCM algorithms built in CT scanners from different vendors, which can significantly affect estimated doses for protocols when TCM is used.^37^ However, our results indicated that CT scanner models do not significantly influence k‐factor individually. Similar results have also been reported in the literature.^36^ As presented in Table 6, compared with k‐factors derived from AAPM Report 96^18^, there was a significant difference, especially for pediatric phantoms. While AAPM Report 96 provides generalized values, they do not account for different patient and scan parameters, which were included in our study. To accommodate those differences and to allow comparison, adjustments in our data were made. To accommodate the lack of patent size factors in ICRP102/AAPM Report 96, we used mean values for k‐factors, and a model without TCM was used for comparison. Differences of over ± 200% supported the significance of patient and scan parameters, which in clinical practice could be even larger, leading to less accurate effective dose estimation, and it should be carefully interpreted and considered as a reference rather than a personalized risk assessment. Relying on the method that systematically underestimates dose can lead to high cumulative radiation exposure, while overestimating might prompt unnecessary “unjustified” lowering of doses that reduces image quality. Because of that, data verification and validation of different software packages against each other and known phantom measurements could help to improve accuracy. At the same time, a comprehensive analysis of these discrepancies could help address the standardization challenges and enableclinical practitioners to better understand the limitations and “over‐simplification” of their chosen tools.

K‐factors in this study, which were calculated based on empirical formulas, could differ from those in other studies using different phantoms with different organ size or location inside the phantom. It is worth saying that these empirical formulas for k‐factor calculations could also be influenced by anatomical differences in real patients or their positioning inside the scanner. All of this could affect effective dose estimation. Hence, more research is still needed.

This study has potential limitations that should be considered in the future. VirtualDose is based on a comprehensive database of Monte Carlo simulation results, but does not explicitly perform Monte Carlo simulations.^7^ However, it was found in a previous study that, fordoses to several organs inside the abdomen, VirtualDose results were within relatively small variations (20%) from Monte Carlo simulations of CT scans on several phantoms.^38^ Second, only one TCM scheme is available. When TCM use is selected, the values of mAs and CTDI_w_ will be approximated based on the entered mAs per rotation, which should be the average mAs per rotation for the entire scan. When TCM is not in use, this is a constant value.^7^ Therefore, the TCM scheme used in the VirtualDose software modulates tube current more similarly to that of GE system, which may lead to some differences among different vendors.

## 5 CONCLUSIONS

This study demonstrates that considerable differences exist in calculated k‐factors, primarily influenced by patient size, scan length, and pediatric age, which have meaningful implications for the accuracy of effective dose estimations in clinical applications. The findings highlight the need to develop a more comprehensive set of k‐factors that incorporate key patient‐specific parameters, which may improve the accuracy of effective dose estimates. Given that CT scanner model differences did not significantly affect k‐factors, the results advocate for adopting universal scanner models to minimize unnecessary complexity in effective dose estimation. With that said, k‐factors should not be used for personalized risk assessment; they are intended for approximate dose comparison and population‐level radiation‐protection purposes.

## AUTHOR CONTRIBUTIONS

Jelena M. Mihailovic, Kai Yang, and Lifeng Yu conceptualize the study. Jelena M. Mihailovic collected and analyzed data. Jelena M. Mihailovic, Renxin Chu, and Kai Yang were responsible for the preparation of the initial manuscript. All authors participated in the discussion of results and revision of the manuscript.

## CONFLICT OF INTEREST STATEMENT

The authors declare no conflicts of interest.

## ETHICS STATEMENT

This research did not involve human or animal subjects and is exempt from ethics committee approval.

## Supporting information




Supporting Information

## Data Availability

The data generated during and/or analyzed during the current study are available from the corresponding author upon reasonable request.

## APPENDIX A

Results from multivariable linear regression analysis for adult and pediatric phantoms without TCM with empirical formulas for k‐factor calculations.

**Table:**

Head (adult)					
Parameters	Slope	Coefficients	Lower 95% CI	Upper 95% CI	p value
Intercept (mSv/mGy×cm)		−0.002216853	−0.003716374	−0.000717332	0.004233708
kV (mSv/mGycm×kV)	0.00001	1.14531E‐05	6.05472E‐06	1.68515E‐05	6.12717E‐05
Phantom Size (BMI) (mSv/mGycm^2^×kg)	0.00001	9.51889E‐06	2.63508E‐06	1.64027E‐05	0.007292698
Gender (mSv/mGy×cm)	0.008	0.00121717	0.000977766	0.001456574	3.10499E‐16
Scan length (mSv/mGy×cm^2^)	−0.001	0.000259964	0.000151996	0.000367932	7.06723E‐06
Vendor (mSv/mGy×cm	−1E‐18	−4.1E‐20	−6.1E‐05	6.07E‐05	1
Adjusted R square (0.595995159)					

$k-factor=-0.0022+1.14e^{-5}\times kV+9.52e^{-6}\times BMI+0.0012\times Gender+0.00026\times Scan\;Length-4.1e^{-2}\times Vendor$

**Table:**

Neck (Adult)					
Parameters	Slope	Coefficients	Lower 95% CI	Upper 95% CI	p value
Intercept (mSv/mGy×cm)		0.011967743	0.010588668	0.013346818	1.6491E‐29
kV (mSv/mGycm×kV)	0.00001	1.08076E‐05	5.9705E‐06	1.56447E‐05	2.66313E‐05
Phantom Size (BMI) (mSv/mGycm^2^×kg)	−0.00002	−1.73012E‐05	−2.34693E‐05	−1.11332E‐05	2.84178E‐07
Gender (mSv/mGy×cm)	0.0007	0.000317801	0.000121755	0.000513847	0.00179913
Scan length (mSv/mGy×cm^2^)	−0.0004	−0.000343206	−0.000439948	−0.000246464	4.35384E‐10
Vendor (mSv/mGy×cm)	0.01E‐20	−2.2E‐20	−5.4E‐05	5.44E‐05	1
*Adjusted R square (0.670276402)*					

$k-factor=0.0119+1.08e^{-5}\times kV-1.73e^{-5}BMI+0.00031\times Gender-0.00034\times Scan\;Length-2.2e^{-20}\times Vendor$

**Table:**

Chest (Adult)					
Parameters	Slope	Coefficients	Lower 95% CI	Upper 95% CI	p value
Intercept (mSv/mGy×cm)		0.020010426	0.016296524	0.023724328	1.91033E‐17
kV (mSv/mGycm×kV)	0.00004	3.85737E‐05	−0.000137928	0.000131401	0.961681985
Phantom Size (mSv/mGycm^2^×kg)	−0.0002	−0.000180586	−0.000189172	−0.000172001	1.79227E‐58
Gender (mSv/mGy×cm)	0.0015	0.001465093	0.001096136	0.00183405	9.19442E‐12
Scan length (mSv/mGy×cm^2^)	−0.0004	−3.26353E‐06	−0.000137928	0.000131401	0.961681985
Vendor (mSv/mGy×cm)	−9E‐18	1.12E‐19	−7.6E‐5	7.5E‐5	7.57E‐5
*Adjusted R square (0.958426529)*					

$k-factor=0.0200+3.86e^{-5}\times kV-0.00018\times BMI+0.0014\times Gender-3.26e^{-6}\times Scan\;Length+1.12e^{-19}\times Vendor$

**Table:**

Abdomen (Adult)					
Parameters	Slope	Coefficients	Lower 95% CI	Upper 95% CI	p value
Intercept (mSv/mGy×cm)		0.026214457	0.022014904	0.030414009	8.79393E‐21
kV (mSv/mGycm×kV)	−0.0003	3.49837E‐05	2.23207E‐05	4.76467E‐05	4.04338E‐07
Phantom Size (mSv/mGycm^2^×kg)	0.000035	−0.000347917	−0.000364064	−0.00033177	2.52858E‐5
Gender (mSv/mGy×cm)	0.0023	0.002170242	0.001698018	0.002642467	2.8421E‐14
Scan length (mSv/mGy×cm^2^)	−0.0007	−0.000127908	−0.000381168	0.000125352	0.318147486
Vendor (mSv/mGy×cm)	3E‐17	1.37E‐19	−0.0.00014231	0.000142	1
*Adjusted R square (0.957049917)*					

$k-factor=0.0262+3.49e^{-5}\times kV-0.00035\times BMI+0.0022\times Gender-0.00012\times Scan\;Length+1.37e^{-19}\times Vendor$

**Table:**

Pelvis (Adult)					
Parameters	Slope	Coefficients	Lower 95% CI	Upper 95% CI	p value
Intercept (mSv/mGy×cm)		0.012748414	0.007058743	0.018438085	2.54007E‐05
kV (mSv/mGycm×kV)	0.00007	3.36557E‐05	1.79344E‐05	4.93771E‐05	5.32602E‐05
Phantom Size (BMI) (mSv/mGycm^2^×kg)	−0.0045	−0.000228507	−0.000248554	−0.00020846	8.70459E‐38
Gender (mSv/mGy×cm)	0.0036	0.0035535	0.002897208	0.004209793	1.49919E‐17
Scan length (mSv/mGy×cm^2^)	−0.0061	−4.02973E‐05	−0.000354725	0.00027413	0.799478707
Vendor (mSv/mGy×cm)	9E‐18	−5.5E‐19	−0.00018	0.000177	1
*Adjusted R square (0.890363186)*					

$k-factor=0.0127+3.37e^{-5}\times kV-0.00023\times BMI+0.0035\times Gender-4.03e^{-5}\times Scan\;Length-5.5^{-19}\times Vendor$

**Table:**

AP (Adult)					
Parameters	Slope	Coefficients	Lower 95% CI	Upper 95% CI	p value
Intercept (mSv/mGy×cm)		0.017907923	0.009704646	0.026111199	3.90231E‐05
kV (mSv/mGycm×kV)	0.000035	3.42019E‐05	2.18766E‐05	4.65271E‐05	3.65139E‐07
Phantom Size (BMI) (mSv/mGycm^2^×kg)	0.0003	−0.000283532	−0.000299249	−0.000267816	4.14921E‐53
Gender (mSv/mGy×cm)	0.0031	0.003055201	0.002379817	0.003730585	5.547E‐14
Scan length (mSv/mGy×cm^2^)	0.0009	−5.26532E‐06	−0.000251771	0.00024124	0.966224386
Vendor (mSv/mGy×cm)	5E‐18	−6.5E‐19	−0.00014	0.000139	1
*Adjusted R square (0.945448051)*					

$k-factor=0.0179+3.42e^{-5}\times kV-0.00028\times BMI+0.003\times Gender-5.26e^{-6}\times Scan\;Length-6.5e^{-19}\times Vendor$

**Table:**

CAP (Adult)					
Parameters	Slope	Coefficients	Lower 95% CI	Upper 95% CI	p value
Intercept (mSv/mGy×cm)		0.026168478	0.015704102	0.036632854	3.40139E‐06
kV (mSv/mGycm×kV)	0.00003	3.30233E‐05	2.39514E‐05	4.20952E‐05	1.88658E‐10
Phantom Size (BMI) (mSv/mGycm^2^×kg)	−0.0002	−0.000236131	−0.000247699	−0.000224563	2.02769E‐57
Gender (mSv/mGy×cm)	0.0024	0.001887843	0.001103941	0.002671746	7.04093E‐06
Scan length (mSv/mGy×cm^2^)	−0.0005	−0.000127208	−0.000308646	5.42303E‐05	0.166953777
Vendor (mSv/mGy×cm)	−4E‐17	−5.1E‐19	−0.0001	0.000102	1
*Adjusted R square (0.956482368)*					

$k-factor=0.0261+3.30e^{-5}\times kV-0.00023\times BMI+0.0018\times Gender-0.00013\times Scan\;Length-5.1e^{-19}\times Vendor$

**Table:**

Head (Pediatric)					
Parameters	Slope	Coefficients	Lower 95% CI	Upper 95% CI	p value
Intercept (mSv/mGy×cm)		0.006944614	0.002709049	0.011180178	0.001944801
kV (mSv/mGycm×kV)	0.000003	2.622E‐06	−3.22955E‐05	3.75395E‐05	0.880206642
Age (mSv/mGycm×years)	−0.0002	−0.000131728	−0.000281071	1.76154E‐05	0.082267463
Gender (mSv/mGy×cm)	−2E‐18	1.91E‐19	−0.00044	0.000438	0.000438
Scan length (mSv/mGy×cm^2^)	−0.0003	−0.000111014	−0.000368828	0.0001468	0.389577291
Vendor (mSv/mGy×cm)	0.1E‐20	−3.4E‐20	−0.00027	0.000268	0.000268
*Adjusted R square (0.783380575)*					

$k-factor=0.0069+2.62e^{-6}\times kV-0.00013\times Age+1.91e^{-19}\times Gender-0.00011\times Scan\;Length-3.4e^{-20}\times Vendor$

**Table:**

Neck (Pediatric)					
Parameters	Slope	Coefficients	Lower 95% CI	Upper 95% CI	p value
Intercept (mSv/mGy×cm)		0.032143171	0.020381137	0.043905205	2.08567E‐06
kV (mSv/mGycm×kV)	0.00003	2.69313E‐05	−6.20799E‐05	0.000115943	0.544549992
Age (mSv/mGycm×years)	−0.0014	−0.000543794	−0.001238213	0.000150624	0.121451113
Gender (mSv/mGy×cm)	−2E‐17	1.48E‐18	−0.001112	0.0011117	0.0011117
Scan length (mSv/mGy×cm^2^)	−0.0033	−0.00210113	−0.003759457	−0.000442802	0.014292342
Vendor (mSv/mGy×cm)	−9E‐18	4.53E‐19	−0.00068	0.000684	0.000684
*Adjusted R square (0.743380575)*					

$k-factor=0.0321+2.69e^{-5}\times kV-0.00054\times Age+1.48e^{-18}\times Gender-0.0021\times Scan\;Length+4.53e^{-19}\times Vendor$

**Table:**

Chest (Pediatric)					
Parameters	Slope	Coefficients	Lower 95% CI	Upper 95% CI	p value
Intercept (mSv/mGy×cm)		0.161342925	0.122942927	0.199742923	1.44217E‐10
kV (mSv/mGycm×kV)	0.00007	7.2409E‐05	−0.000211503	0.000356321	0.609273452
Age (mSv/mGycm×years)	−0.006	0.002316229	−0.000996583	0.00562904	0.165492623
Gender (mSv/mGy×cm)	−2E‐17	3.59E‐18	−0.00356	0.003536	1
Scan length (mSv/mGy×cm^2^)	−0.0064	−0.008612441	−0.011950256	−0.005274626	5.67653E‐06
Vendor (mSv/mGy×cm)	7E‐17	2.84E‐18	−0.00218	0.002181	1
*Adjusted R square (0.844394245)*					

$k-factor=0.1613+7.24e^{-5}\times kV+0.0023\times Age+3.59e^{-18}\times Gender-0.0119\times Scan\;Length+2.84e^{-18}\times Vendor$

**Table:**

Abdomen (Pediatric)					
Parameters	Slope	Coefficients	Lower 95% CI	Upper 95% CI	p value
Intercept (mSv/mGy×cm)		0.160705874	0.110649396	0.210762352	8.87515E‐08
kV (mSv/mGycm×kV)	0.00007	6.77796E‐05	−0.000236647	0.000372206	0.655335156
Age (mSv/mGycm×years)	−0.0045	0.001372366	−0.001594553	0.004339285	0.355695383
Gender (mSv/mGy×cm)	−2E‐17	−2.6E‐18	−0.00381	0.00382	1
Scan length (mSv/mGy×cm^2^)	−0.0061	−0.007745154	−0.011493827	−0.003996481	0.000152573
Vendor (mSv/mGy×cm)	−7E‐17	−3.1E‐18	−0.00234	0.002339	1
*Adjusted R square (0.73142786)*					

$k-factor=0.1607+6.77e^{-5}\times kV-0.0014\times Age-2.6^{-18}\times Gender-0.0077\times Scan\;Length-3.1e^{-18}\times Vendor$

**Table:**

Pelvis (Pediatric)					
Parameters	Slope	Coefficients	Lower 95% CI	Upper 95% CI	p value
Intercept (mSv/mGy×cm)		0.068285094	0.053452241	0.083117947	1.19103E‐11
kV (mSv/mGycm×kV)	0.00004	4.38288E‐05	−6.31467E‐05	0.000150804	0.412787375
Age (mSv/mGycm×years)	−0.0028	−0.000235707	−0.004609142	−0.001706232	7.68999E‐05
Gender (mSv/mGy×cm)	0.1E‐19	−3.7E‐18	−0.00134	0.001342	1
Scan length (mSv/mGy×cm^2^)	−0.0024	−0.003157687	−0.004609142	−0.001706232	7.68999E‐05
Vendor (mSv/mGy×cm)	6E‐17	−3.1E‐18	−0.00082	0.000822	1
*Adjusted R square (0.887427207)*					

$k-factor=0.0682+4.38e^{-5}\times kV-0.00023\times Age-3.7e^{-18}\times Gender-0.0032\times Scan\;Length-3.1e^{-18}\times Vendor$

**Table:**

AP (Pediatric)					
Parameters	Slope	Coefficients	Lower 95% CI	Upper 95% CI	p value
Intercept (mSv/mGy×cm)		0.155561536	0.123976271	0.1871468	1.71715E‐12
kV (mSv/mGycm×kV)	0.00006	5.6203E‐05	−0.00012765	0.000240056	0.540407321
Age (mSv/mGycm×years)	−0.0042	0.003395647	−0.006492292	−0.003591228	1.54663E‐08
Gender (mSv/mG×ycm)	1E‐16	1.06E‐16	−0.00231	0.00231	1
Scan length (mSv/mGy×cm^2^)	−0.0029	−0.00504176	−0.006492292	−0.003591228	1.54663E‐08
Vendor (mSv/mGy×cm)	2E‐16	8.28E‐18	−0.00141	0.00141	1
*Adjusted R square (0.870049523)*					

$k-factor=0.1556+5.62e^{-5}\times kV+0.0034\times Age+1.06e^{-16}\times Gender-0.005\times Scan\;Length+8.28e^{-18}\times Vendor$

**Table:**

CAP (Pediatric)					
Parameters	Slope	Coefficients	Lower 95% CI	Upper 95% CI	p value
Intercept (mSv/mGy×cm)		0.176494624	0.163624156	0.189365092	3.66533E‐28
kV (mSv/mGycm×kV)	0.00006	6.18789E‐05	−2.02041E‐05	0.000143962	0.135573412
Age (mSv/mGycm×years)	−0.0044	0.005049647	0.004113362	0.005985932	1.12692E‐13
Gender (mSv/mGy×cm)	−4E‐17	1.03E‐17	−0.0013	0.0013	1
Scan length (mSv/mGy×cm^2^)	−0.0021	−0.004238691	−0.004643911	−0.00383347	1.18596E‐23
Vendor (mSv/mGy×cm)	−4E‐17	7.89E‐18	−0.00063	0.00063	1
*Adjusted R square (0.978010791)*					

$k-factor=0.1764+6.18e^{-5}\times kV+0.005\times Age+1.03e^{-17}\times Gender-0.0042\times Scan\;Length+7.89e^{-18}\times Vendor$

Results from multivariable linear regression analysis for adult and pediatric phantoms with TCM and empirical formulas for k‐factor calculations.

**Table:**

Head (Adult)					
Parameters	Slope	Coefficients	Lower 95% CI	Upper 95% CI	p value
Intercept (mSv/mGy×cm)		0.000166228	−5.02767E‐05	0.000382733	0.1305848
kV (mSv/mGycm×kV)	0.000009	9.4315E‐06	8.65206E‐06	1.02109E‐05	1.0665E‐39
Phantom Size (mSv/mGycm^2^×kg)	−0.000002	−1.9031E‐06	−2.89701E‐06	−9.09202E‐07	0.00026464
Gender (mSv/mGy×cm)	0.0006	0.000791117	0.000756551	0.000825683	1.8469E‐61
Scan length (mSv/mGy×cm^2^)	−0.0001	0.000128235	0.000112647	0.000143824	5.4915E‐28
Vendor (mSv/mGy×cm)	5E‐18	−5.7E‐20	−9.5E‐06	9.5E‐06	1
*Adjusted R square (0.971592152)*					

$k-factor=0.0002+9.43e^{-6}\times kV-1.90e^{-6}\times BMI+0.00079\times Gender+0.00013\times Scan\;Length-5.7e^{-20}\times Vendor$

**Table:**

Neck (Adult)					
Parameters	Slope	Coefficients	Lower 95% CI	Upper 95% CI	p value
Intercept (mSv/mGy×cm)		0.009462001	0.008283346	0.010640656	2.6421E‐27
kV (mSv/mGycm×kV)	0.00002	2.08322E‐05	1.66981E‐05	2.49663E‐05	4.704E‐16
Phantom Size (mSv/mGycm^2^×kg)	0.000007	7.16123E‐06	1.8896E‐06	1.24329E‐05	0.00834373
Gender (mSv/mGy×cm)	0.0005	7.3088E‐05	−9.4467E‐05	0.000240643	0.38822582
Scan length (mSv/mGy×cm^2^)	−0.0004	−0.000358122	−0.000440805	−0.00027544	3.2978E‐13
Vendor (mSv/mGy×cm)	5E‐18	−5.6E‐20	−4.8E‐05	4.83E‐05	1
*Adjusted R square (0.723317903)*					

$k-factor=0.0095+2.08e^{-5}\times kV+7.161e^{-6}BMI+7.31e^{-5}\times Gender-0.00036\times Scan\;Length-5.6e^{-20}\times Vendor$

**Table:**

Chest (Adult)					
Parameters	Slope	Coefficients	Lower 95% CI	Upper 95% CI	p value
Intercept (mSv/mGy×cm)		0.024482787	0.021490607	0.027474967	7.7615E‐28
kV (mSv/mGycm×kV)	0.00003	2.53591E‐05	1.99343E‐05	3.07838E‐05	1.3676E‐14
Phantom Size (mSv/mGycm^2^×kg)	−0.0002	−0.000173471	−0.000180389	−0.000166554	1.0308E‐64
Gender (mSv/mGy×cm)	0.002	0.001802555	0.001505298	0.002099813	4.2718E‐20
Scan length (mSv/mGy×cm^2^)	−0.0006	−9.53355E‐05	−0.00020383	1.31593E‐05	0.08422991
Vendor (mSv/mGy×cm)	7E‐17	−2.9E‐20	−0.00072	0.000724	1
*Adjusted R square (0.971929037)*					

$k-factor=0.0245+2.54^{-5}\times kV-0.0002\times BMI+0.0018\times Gender-9.53^{-5}\times Scan\;Length-2.9^{-20}\times Vendor$

**Table:**

Abdomen (Adult)					
Parameters	Slope	Coefficients	Lower 95% CI	Upper 95% CI	p value
Intercept (mSv/mGy×cm)		0.026391034	0.022297649	0.03048442	1.4517E‐21
kV (mSv/mGycm×kV)	0.00004	3.55564E‐05	2.32135E‐05	4.78992E‐05	1.5037E‐07
Phantom Size (mSv/mGycm^2^×kg)	−0.0004	−0.000374419	−0.000390158	−0.00035868	7.8221E‐63
Gender (mSv/mGy×cm)	0.0022	0.002097428	0.001637141	0.002557714	4.0799E‐14
Scan length (mSv/mGy×cm^2^)	0.0006	−9.18144E‐05	−0.000338672	0.000155043	0.46163973
Vendor (mSv/mGy×cm)	−9E‐18	−2.7E‐19	−0.00014	0.000139	1
*Adjusted R square (0.963984877)*					

$k-factor=0.0263+3.55e^{-5}\times kV-0.00037\times BMI+0.0021\times Gender-9.18e^{-5}\times Scan\;Length-2.7e^{-19}\times Vendor$

**Table:**

Pelvis (Adult)					
Parameters	Slope	Coefficients	Lower 95% CI	Upper 95% CI	p value
Intercept (mSv/mGy×cm)		0.012810717	0.006865714	0.01875572	4.8025E‐05
kV (mSv/mGycm×kV)	0.00003	3.35915E‐05	1.71646E‐05	5.00184E‐05	0.00010647
Phantom Size (mSv/mGycm^2^×kg)	−0.0002	−0.000230464	−0.00025141	−0.000209517	1.139E‐36
Gender (mSv/mGy×cm)	0.0037	0.003650632	0.002964888	0.004336377	3.4424E‐17
Scan length (mSv/mGy×cm^2^)	−0.0011	−4.11946E‐05	−0.000369732	0.000287343	0.80372695
Vendor (mSv/mGy×cm)	−7E‐17	4.98E‐20	−0.00072	0.00072	1
*Adjusted R square (0.884202731)*					

$k-factor=0.0128+3.36e^{-5}\times kV-0.00023\times BMI+0.0036\times Gender-4.12^{-5}\times Scan\;Lenght+4.98e^{-20}\times Vendor$

**Table:**

AP (Adult)					
Parameters	Slope	Coefficients	Lower 95% CI	Upper 95% CI	p value
Intercept (mSv/mGy×cm)		0.00891375	−0.002818749	0.020646249	0.13460328
kV (mSv/mGycm×kV)	0.00005	5.03462E‐05	3.27183E‐05	6.79741E‐05	1.8514E‐07
Phantom Size (mSv/mGycm^2^×kg)	−0.0003	−0.000308205	−0.000330683	−0.000285727	8.6802E‐44
Gender (mSv/mGy×cm)	0.0027	0.003229988	0.00226404	0.004195936	2.6981E‐09
Scan length (mSv/mGycm^2^)	−0.0007	0.000236846	−0.000115711	0.000589404	0.18521052
Vendor (mSv/mGy×cm)	2E‐17	1.42E‐19	−0.00072	0.00072	1
*Adjusted R square (0.906291036)*					

$k-factor=0.0089+5.03e^{-5}\times kV-0.0003\times BMI+0.0032\times Gender+0.00023\times Scan\;Lenght+1.42e^{-19}\times Vendor$

**Table:**

CAP (Adult)					
Parameters	Slope	Coefficients	Lower 95% CI	Upper 95% CI	p value
Intercept (mSv/mGy×cm)		0.024812883	0.01093855	0.038687216	0.00061862
kV (mSv/mGycm×kV)	0.00004	4.39684E‐05	3.19403E‐05	5.59965E‐05	1.6421E‐10
Phantom Size (mSv/mGycm^2^×kg)	−0.0003	−0.000324154	−0.000339492	−0.000308816	1.2129E‐58
Gender (mSv/mGy×cm)	0.0028	0.002413627	0.00137428	0.003452975	1.3682E‐05
Scan length (mSv/mGy×cm^2^)	−0.0005	−8.88249E‐05	−0.000329387	0.000151737	0.46488033
Vendor (mSv/mGy×cm)	9E‐18	5.02E‐20	−0.00063	0.00063	1
*Adjusted R square (0.957629755)*					

$k-factor=0.0248+4.39e^{-5}\times kV-0.00032\times BMI+0.0024\times Gender-8.88e^{-5}\times Scan\;Length+5.02e^{-2}\times Vendor$

**Table:**

Head (Pediatric)					
Parameters	Slope	Coefficients	Lower 95% CI	Upper 95% CI	p value
Intercept (mSv/mGy×cm)		0.006719869	0.005326577	0.00811316	3.1584E‐12
kV (mSv/mGycm×kV)	0.00002	2.27948E‐05	1.29275E‐05	3.2662E‐05	3.2673E‐05
Age(mSv/mGycm×years)	−0.0002	−0.000147215	−0.000192136	−0.000102295	5.7128E‐08
Gender (mSv/mGy×cm)	1E‐20	−3.8E‐19	−00012	0.00012	1
Scan length (mSv/mGy×cm^2^)	−0.0005	−0.000249716	−0.000349279	−0.000150152	9.0915E‐06
Vendor (mSv/mGy×cm)	−9E‐18	−3E‐19	−7.6E‐05	7.58E‐05	1
*Adjusted R square (0.878409502)*					

$k-factor=0.0067+2.28e^{-5}\times kV-0.00015\times Age-3.8e^{-19}\times Gender-0.00025\times Scan\;Length-3e^{-19}\times Vendor$

**Table:**

Neck (Pediatric)					
Parameters	Slope	Coefficients	Lower 95% CI	Upper 95% CI	p value
Intercept (mSv/mGy×cm)		0.027723497	0.015906258	0.039540735	2.5954E‐05
kV (mSv/mGycm×kV)	0.00003	3.43459E‐05	−5.5083E‐05	0.000123775	0.44242358
Age (mSv/mGycm×years)	−0.0012	−0.00060715	−0.001304828	9.05278E‐05	0.08629876
Gender (mSv/mGy×cm)	6E‐18	1.58E‐18	−0.00112	0.0012	1
Scan length (mSv/mGy×cm^2^)	−0.003	−0.001653408	−0.003319519	1.27028E‐05	0.05169094
Vendor (mSv/mGy×cm)	9E‐18	1.03E‐18	−0.00069	0.00069	1
*Adjusted R square (0.70269698)*					

$k-factor=0.0277+3.43e^{-5}\times kV-0.0006\times Age+1.58e^{-18}\times Gender-0.00165\times Scan\;Length+1.03e^{-18}\times Vendor$

**Table:**

Chest (Pediatric)					
Parameters	Slope	Coefficients	Lower 95% CI	Upper 95% CI	p value
Intercept (mSv/mGy×cm)		0.163956369	0.124632488	0.20328025	1.7674E‐10
kV (mSv/mGycm×kV)	0.00006	6.26294E‐05	−0.00022943	0.000354689	0.66723236
Age (mSv/mGycm×years)	−0.0061	0.002158314	−0.001231461	0.005548089	0.20570412
Gender (mSv/mGy×cm)	1E‐19	6.33E‐18	−0.00366	0.00366	1
Scan length (mSv/mGy×cm^2^)	−0.0064	−0.008533944	−0.011935033	−0.005132856	9.0276E‐06
Vendor (mSv/mGy×cm)	−4E‐17	4.14E‐18	−0.00224	0.00224	1
*Adjusted R square (0.841290503)*					

$k-factor=0.1639+6.26e^{-5}\times kV+0.0022\times Age+6.33e^{-18}\times Gender-0.0085\times Scan\;Length+4.14e^{-1}\times Vendor$

**Table:**

Abdomen (Pediatric)					
Parameters	Slope	Coefficients	Lower 95% CI	Upper 95% CI	p value
Intercept (mSv/mGy×cm)		0.146057306	0.097122132	0.194992479	3.9507E‐07
kV (mSv/mGycm×kV×)	0.00007	6.54793E‐05	−0.000232128	0.000363086	0.6591349
Age (mSv/mGy×years)	−0.0049	−0.000243086	−0.003143544	0.002657371	0.86642687
Gender (mSv/mGy×cm)	1E‐19	1.27E‐18	−0.0037	0.0037	1
Scan length (mSv/mGy×cm^2^)	−0.0065	−0.006196203	−0.009860903	−0.002531503	0.00145126
Vendor (mSv/mGy×cm)	−4E‐17	−5.2E‐19	−0.00229	0.00229	1
*Adjusted R square (0.763318787)*					

$k-factor=0.1461+6.55e^{-5}\times kV-0.00024\times Age+1.27e^{-18}\times Gender-0.0062\times Scan\;Length-5.2e^{-19}\times Vendor$

**Table:**

Pelvis (Pediatric)					
Parameters	Slope	Coefficients	Lower 95% CI	Upper 95% CI	p value
Intercept (mSv/mGy×cm)		0.068932771	0.053833403	0.084032139	1.506E‐11
kV (mSv/mGycm×kV)	0.00004	4.23307E‐05	−6.65669E‐05	0.000151228	0.43694484
Age (mSv/mGycm×years)	−0.0029	−0.000458619	−0.00169373	0.000776492	0.45760132
Gender (mSv/mGy×cm)	2E‐17	−3E‐18	−0.00137	0.00137	1
Scan length (mSv/mGy×cm^2^)	−0.0036	−0.003038086	−0.00451562	−0.001560551	0.00016226
Vendor (mSv/mGy×cm)	7E‐17	−2.7E‐18	−0.00084	0.000843	1
*Adjusted R square (0.891805279)*					

$k-factor=0.0689+4.23e^{-5}\times kV-0.00046\times Age-3e^{-18}\times Gender-0.0030\times Scan\;Length-2.7e^{-18}\times Vendor$

**Table:**

AP (Pediatric)					
Parameters	Slope	Coefficients	Lower 95% CI	Upper 95% CI	p value
Intercept (mSv/mGy×cm)		0.156718791	0.124557449	0.188880134	2.3437E‐12
kV (mSv/mGycm×kV×)	0.00005	4.78853E‐05	−0.000139321	0.000235091	0.60822639
Age (mSv/mGycm×years)	−0.0043	0.003144224	0.000867094	0.005421354	0.00799193
Gender (mSv/mGy×cm)	5E‐17	8.97E‐18	−0.00235	0.00236	1
Scan length (mSv/mGy×cm^2^)	−0.0003	−0.004946939	−0.006423927	−0.003469951	3.5512E‐08
Vendor (mSv/mGy×cm)	1E‐16	6.21E‐18	−0.001444	0.001439	1
*Adjusted R square (0.870176279)*					

$k-factor=0.1567+4.79e^{-5}\times kV+0.0031\times Age+8.97e^{-18}\times Gender-0.0049\times Scan\;Length+6.21e^{-18}\times Vendor$

**Table:**

CAP (Pediatric)					
Parameters	Slope	Coefficients	Lower 95% CI	Upper 95% CI	p value
Intersept (mSv/mGycm)		0.175673949	0.160189889	0.191158009	5.4695E‐25
kV (mSv/mGycm×kV)	0.00006	6.1034E‐05	−3.77174E‐05	0.000159786	0.21904214
Age (mSv/mGycm×years)	−0.0045	0.004811814	0.003685399	0.005938229	9.2813E‐11
Gender (mSv/mGy×cm)	1E‐18	1.08E‐17	−0.00124	0.001239	1
Scan length (mSv/mGy×cm^2^)	−0.0022	−0.004177968	−0.004665476	−0.00369046	1.8694E‐20
Vendor (mSv/mGy×cm)	2E‐17	8.47E‐18	−0.00076	0.000759	1
*Adjusted R square (0.967694734)*					

$k-factor=0.1757+6.10e^{-5}\times kV+0.0048\times Age+1.08e^{-18}\times Gender-0.0042\times Scan\;Length+8.47e^{-18}\times Vendor$
