# Supplementary material for: Quantifying variation in DLP‐ to‐ effective‐dose conversion factors in CT using a Monte Carlo‐based dose‐estimation software
Source: J Appl Clin Med Phys. 2026 Aug 30;27(9):e70724. doi: 10.1002/acm2.70724 (PMC13527227; doi:10.1002/acm2.70724)
Supplement: Supplementary file 1 [file ACM2-27-e70724-s001.docx]

Figure S1. Violin plot of the conversion factors for the adult phantoms across the whole study. The range of the distribution is largest for the abdomen (blue), followed by AP (light green), CAP ( dark gray), chest, and pelvis (dark green and purple, respectively) exams (A). Similar is seen for studies that use TCM (B).

Figure S2. Violin plot of the conversion factors for the pediatric phantoms across the whole study. The range of the distribution is largest for the chest (dark green), followed by the abdomen (blue), pelvis (purple), AP (light green), and CAP (dark gray) exams (A). Similar is seen for studies that use TCM (B).
